# Editorial on the topical issue of charged species in bulk and at interfaces

**DOI:** 10.1140/epje/s10189-025-00475-6

**Published:** 2025-03-28

**Authors:** Emanuela Bianchi, Kyongok Kang

**Affiliations:** 1 Institute fur Theoretische Physik, Technische Universitat Wien, Vienna, Austria; 2 Forschungszentrum Jülich, Institute of Biological Information Processing, IBI-4: Biomacromolecular Systems and Processes, 52428 Jülich, Germany

## Abstract

**Abstract:**

The topical issue titled “Charged Species in Bulk and at Interfaces: Interaction, Mobility, Transport, and Regulation” is based on contributions from speakers at three CECAM workshops held in 2016, 2018, and 2022. In addition, this editorial is also intended to express our sincere appreciation to our senior co-organizers, Prof. Jan K. G. Dhont (FZJ, Germany) and Prof. Gerhard Kahl (TU Wien, Austria), for their invaluable contributions.

**Graphical Abstract:**

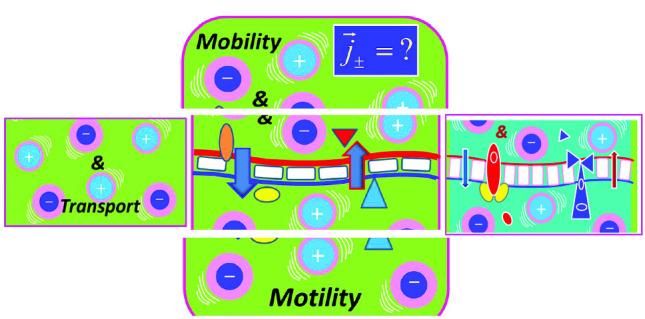

## Introduction

The topical issue titled “Charged Species in Bulk and at Interfaces: Interaction, Mobility, Transport, and Regulation” is derived from a series of three CECAM workshops held in 2016, 2018, and 2022. The aim of this issue is to advance our understanding of the complex phenomena surrounding charged species in various environments from a fundamental perspective, with some excursions into practical applications. The issue addresses the characterization of the interactions between a broad variety of charged species as well as of the complex macroscopic behavior arising from these interactions. Modeling pair interactions between highly charged macromolecules and surfaces resulting from (possibly mobile) charges presents significant challenges; equally challenging is understanding how these interactions govern self-assembly, assembly kinetics, transport in dense systems, response to external electric fields, and regulatory mechanisms. The following three subtopics are covered: (i) fundamental theoretical and simulation challenges in (macro-)molecular electrostatics, (ii) electrostatic interactions in biological processes/systems, and (iii) self-assembly and transport of charged (macro-)molecules. Each contribution highlights different aspects of these themes, offering an overview of the current state of knowledge and ongoing research efforts.

**Fig. 1:**
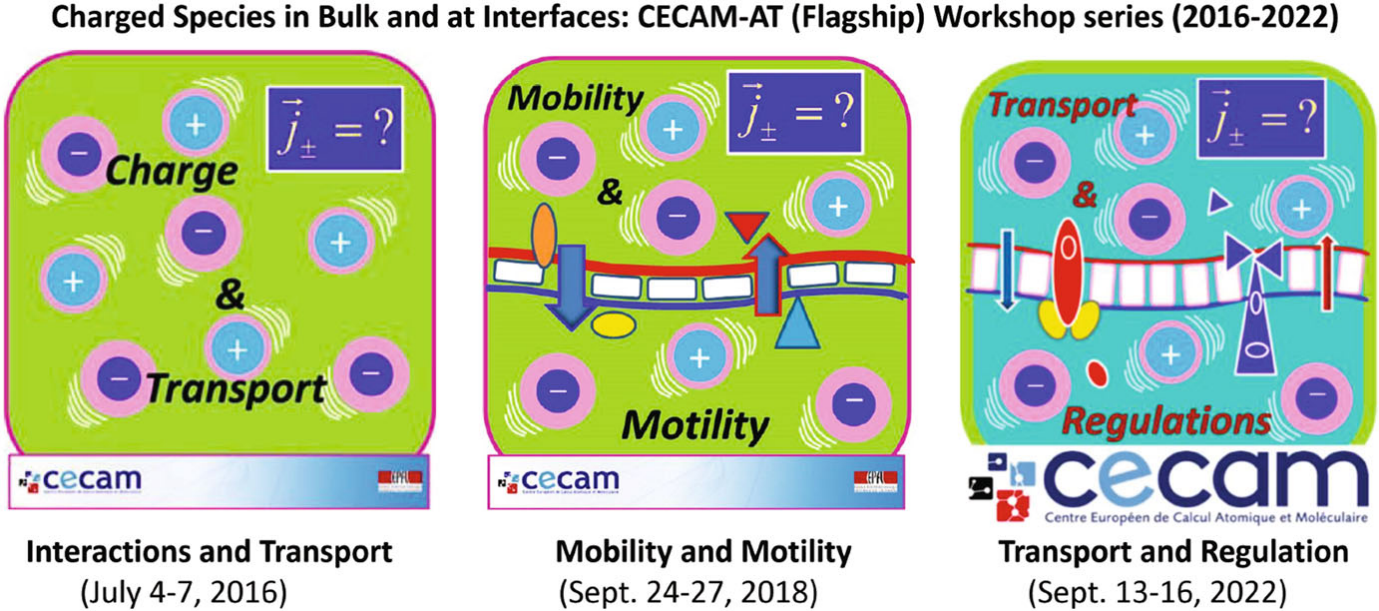
The logos for the CECAM workshops “Charged Species in Bulk and Interfaces”: the first for “Interactions and Transport,” the second for “Mobility and Motility,” followed by the third “Transport and Regulation”

In particular, the transport of charged species, both in bulk and at interfaces, has garnered significant attention due to its fundamental and technological implications. The role of electrostatics and dynamics in biological systems—spanning a wide range of length scales—has become a rapidly growing area of research. At the molecular level, the ordering of water molecules is crucial for the mass transport of ions through confined geometries. This structural ordering within hydration layers also interplays with the electrostatic forces between charged (bio-)macromolecules in general.

Throughout our series of workshops, several key topics were explored, including the confinement-induced structure of water, the hydration of small charged species in bulk, and the interfacial friction between planar and nanoscopic (smectic) layers lubricated by water [1, 2]. Confinement has a significant impact, e.g., on ion correlations and electroosmotic flow, particularly for small proteins that can bridge colloids [3], as well as on the assembly of negatively charged colloids with positively charged polar caps near planar surfaces [4]. Additionally, the confinement-induced structure of water plays a crucial role in processes such as membrane desalination and osmotic power harvesting. There is also a qualitative difference in the degree of ionic hydration for various ions near the air–water interface [5]. Moreover, the deswelling behavior and transport properties of microgel particles are strongly influenced by the release and capture of ions confined within the microgel [6].

The interplay between water structure and electrostatics is fundamental to understanding macromolecular behavior, particularly in biological systems. Charge regulation in macromolecules—whether occurring in equilibrium or driven by external fields—can significantly alter their interactions, often leading to changes in their phase behavior. A striking example of this is seen in the bacteriophage PP7 capsids [7, 8] and DNA viruses (fd) [9], where pH and salt concentration regulate the dissociation equilibrium of amino acids, modulating electrostatic interactions. Another critical factor influencing macromolecular behavior is ionic-strength-dependent osmotic pressure [10], which has been studied using fluorescence-based experimental techniques to visualize small osmotic differences in these systems. In addition to charge regulation and complex charge distributions, shape and size also play significant roles in phenomena such as single-cell motility and the dynamics and structure of complex, crowded environments [11, 12]. Despite these exemplifying advancements in the field, the regulation of charges and the effects of complex charge distributions remain only partially understood, underscoring the need for further investigations into phase behavior.

The papers featured in this CECAM-organized topical issue of EPJE include:

- Computational approaches to lipid-based nucleic acid delivery systems (Giovanni Settanni)
- Effective interactions and phase behavior of protein solutions in the presence of hexamine cobalt(III) chloride (Maximilian D. Senft, Ralph Maier, Anusha Hiremath, Fajun Zhang, and Frank Schreiber)
- Phase transitions of ionic fluids in nanoporous electrodes (Ayeh Emrani, Clifford E. Woodward, and Jan Forsman)
- Effect of repulsive interaction and initial velocity on collective motion process (I. Tarras, A. Eddakoun, A. Hader, S. Moushi, I. Bakassi, R. Et Touizi, I. Achik, M. Eddahby, A. El Bachiri, and Y. Boughaleb)
- A microfluidic platform for the synthesis of polymer and polymer-protein-based protocells (Jessica Ann O’Callaghan, Neha P. Kamat, Kevin B. Vargo, Rajarshi Chattaraj, Daeyeon Lee, and Daniel A. Hammer)
- A hard sphere model for single-file water transport across biological membranes (Gerald S. Manning)
- Local electroneutrality breakdown for electrolytes within varying-section nanopores (Paolo Malgaretti, Ignacio Pagonabarraga, and Jens Harting)
- Ions and dipoles in electric field: nonlinear polarization and field-dependent chemical reaction (Akira Onuki)

In the following, we give an overview of the content of the three CECAM workshops, summarizing the key topics in sections 2, 3, and 4, and presenting the list of participants along with their affiliations in section 5.

## Interactions and transport (2016)

The first workshop focused on the interactions and transport properties of charged species, from small ions, electrons, and protons to larger systems like colloids, synthetic macromolecules, proteins, DNA, and even living cells. Electrostatic interactions were explored in the context of both biological systems and synthetic materials, including charged patchy colloids, inverse micelles, charge-responsive microgels, and ionic liquids. As far as transport properties were concerned, key areas of interest included charge transport through ion channels, the development of tunable, ion-concentration-dependent bio-hydrogel devices, electrokinetic processes at water/oil interfaces (both in the absence and presence of external electric fields), electrochemical biosensors, and chemical ion filtration through various porous membranes. Additional topics included the influence of polar effects in liquid crystals and the application of high-resolution functional MRI (7 Tesla) to study the connectivity of human nerve cells.

Within this framework, many different topics were addressed, as detailed in the following. An ionic-chemical–mechanical model for actin–myosin ATPase cycle in muscle contraction is proposed, including the hydrolysis of bound ATP, where actin filaments serve as anionic polyelectrolytes surrounded by a sheet of condensed counterions (by G. Manning). Counterion-induced swelling of ionic microgels and microcapsules, where a new statistical notion of “dynamic equilibrium” is introduced for both electrostatic contributions and the pressure differences across the periphery of a permeable macroion (by A. Denton). A new design of soft robotic actuators is discussed, where ion transport and redistribution, as well as electrostatic crosslinking and hydrogel-based circuits, play an important role (by O. Velev). It was demonstrated that local charge density fluctuations, excluding long-range integrations, can be effectively studied using numerical sampling methods that go beyond the classic Poisson–Boltzmann approach (by H. Orland). Simulation results were presented for proton migration along membranes involved in cellular energy homeostasis and proton-coupled transport, focusing on ion permeation through membrane channels over time scales ranging from 0.1 fs to 75 ps, and within the energy window of 6.2$-$8.7 RT (by V. Calandrini). Protein structure and dynamics were explored through CRYO-EM, where individual 3D density reconstructions are obtained despite the large-scale conformational variances of macromolecules. Specifically, cyclic-nuclear binding domains (3–4 Å thick) were studied as an example for both open (-cAMP) and closed (+cAMP) states with voltage control (by G. Schroeder). An asymmetric restricted primitive model of molten salts and ionic liquids was presented (by J. Forsman). Ion-pairing and structural transitions at ionic liquid interfaces, with film thicknesses ranging from 5 to 20 nm at room temperature, were studied under the influence of a planar electrode electric field (5 mV, 38 Hz), which imposed a constant potential (by B. Rotenberg). Ion transport in ionic liquids was also examined through a Poisson–Nernst–Planck-type equation (by A. Lee). Charge transport by inverse micelles in nonpolar media was explored, with an unprecedented level of accuracy, capable of detecting a single elementary charge-an approach akin to a “Millikan experiment.” Additionally, a theoretical framework was proposed to explain the observed phenomena (by K. Neyts). The behavior of inverse patchy systems consisting of heterogeneously charged particles was examined, showing that they form layers due to the interplay between directional attractive as well as repulsive interactions (by E. Bianchi). The phase behavior of lactoferritin solutions was found to be strongly influenced by directional-patchy attractions, Coulomb repulsion, and van der Waals attraction (by M. Oskolkova). Similarly, the phase behavior of lysozyme suspensions was modeled using a DLVO potential combined with patchy hydrophobic interactions, yielding excellent agreement with experimental results (by C. Gögelein). Charged colloids in bulk and near oil–water interfaces, where the dielectric constants differed significantly on both sides, were examined using Poisson–Boltzmann theory (by R. van Roij). The self-assembly of charged colloids immersed in binary electrolytes was simulated using multicomponent Lattice–Boltzmann and capillary-driven link-flux methods, with the influence of a magnetic field (by J. Harting). The molecular ordering of chiral liquid crystals was shown to be switchable between states by an external field, making them suitable for fast-switching flexoelectric sensors (by A. Eremin). Electrochemical biosensors, based on high-resolution porous material membranes with pore sizes ranging from 2 to 50 nm, were optimized for selectivity and sensitivity in their filtering properties, with active and conductive materials that provided fast responses and high sensitivity (by D.H. Kim). Inhomogeneous ion transfer due to ion condensation in soggy sand electrolyte solutions was found to have potential applications as additives for secondary batteries (by H. Washizu). A fundamental theoretical study of charge distribution in two interacting parallel plates, with structural variability of charge carriers, revealed non-conventional phase transitions and exotic phases (by E. Trizac). A super-resolution human brain tractography, employing specialized magnetic resonance imaging (MRI) in combination with computer-based diffusion tensor imaging (DTI), revealed that the recognition of languages and emotions is linked to specific fiber-structural order and fiber connectivity (by Z.H. Cho), highlighting how the development of techniques to image ion distributions could prove invaluable for the early diagnosis of brain diseases. Molecular dynamics simulations and neutron scattering experiments were used to investigate heterogeneous water dynamics in soft confinements, specifically at the nanometer scale, including within surfactant lamellae and micelles (by S. Hanot).

## Mobility and motility of macromolecular systems (2018)

The second workshop on the electrostatics of charged species focused in particular on three major themes.

### Fundamental theoretical and simulation challenges in macromolecular electrostatics

The collective- and self-diffusion in suspensions of charge-stabilized colloidal spheres was investigated, taking into account hydrodynamic interactions through time-dependent dynamics simulations. Notably, the comparison of simulations allowed for a critical evaluation of the accuracy of existing theories (G. Nägele). The validity of coarse-graining methods for simulating ionic microgel particles (A. Denton) and dendrimer-like DNA molecules (C. Jochum) was also examined, with a focus on the statistical mechanical theory of effective electrostatic interactions. The limitations of continuum models for the dielectric response at interfaces were highlighted in fully atomistic simulations of interacting charged surfaces and ions in water (R. Netz). These simulations leveraged novel molecular dynamics techniques to handle ion hydration and the polarizability of the hydration layer. The thermal equilibrium of confined counterions in between the weak- and the strong-coupling limits was also examined (E. Trizac) as was the pressure-induced liquid flow through narrow channels with charged walls (B. Werkhoven). Finally, the electrical charge of surfaces in contact with surfactant-doped nonpolar liquids, relevant for applications like electronic ink displays and liquid toner printing, was discussed (F. Strubbe).

### Electrostatics in biological and neuronal processes/systems.

The dynamics of ion transport through membranes was examined via molecular dynamics simulations, specifically in the context of excitatory amino acid transporters (EAATs) which act as anion-selective channels (J.-P. Machtens). Protein aggregation, including pathways to crystallization, gelation, and amorphous aggregation, was studied in presence of multivalent ions (F. Schreiber). The complex behavior shown by droplets of recombinant coacervating charged proteins was discussed (D. Hammer). A molecular explanation was offered for the observation that the aggregation of Amyloid-$\beta$ peptide into oligomers, fibrils, and plaques is influenced by the surrounding ionic environment (A. Horn). The results of atomistic simulations on the diffusion of receptors within membranes provided insights into complex post-synaptic signaling cascades (V. Calandrini). Molecular simulation studies on the binding of DNA and RNA to proteins and small molecules considered the effects of electrostatic interactions on these processes (P. Carloni).

### Self-assembly and transport in synthetic macromolecular and nanostructured systems.

The preferential adsorption of ions at the interface of water–oil mixtures was shown to create an extended electric double layer, which in turn reduces surface tension and drives mesophase formation (A. Onuki). The kinetics of dissolved ions were studied to understand the processes of electrowetting in nanodrops and the breakup of both charged and neutral droplets (J. Harting). The complex electrostatic interactions between surfactant micellar systems—comprising monomers, counterions, and additives—were examined in order to tune the molecular arrangements within micelles and control their flow properties (P. Fisher). Furthermore, self-assembly behaviors were investigated, including the interaction of negatively charged colloids with positively charged polar caps near planar surfaces (E. Bianchi) and the design of spherical microgels that mimic “lock-and-key” self-assembly via bowl-shaped particles (J. Crassous). The principles of ionic mobility were explored in connection with active particle propulsion under electric fields (O. Velev), while the formation of helical nanofilaments using organo-ferrogels in magnetic fields was also discussed (A. Eremin). The structural evolution of growing clusters during cement hydration was examined taking into account electrostatic interactions (J. Dobnikar), alongside studies on the fatigue properties of carbon black in composite materials (C. Gögelein).

## Transport and regulation (CECAM workshop in 2022)

The most recent workshop in the series addressed the transport and regulation of charged species, with a particular emphasis on confined environments, such as nanopores, and more complex and biologically relevant systems where charge distribution and regulation are pivotal. Key themes included charge regulation of macromolecules, both in equilibrium and induced by external fields and the behavior of charged species in highly concentrated electrolytes. Discussions also touched on electrostatic interactions at biological membranes and the electro-permeability of lipid systems, which are critical for understanding cellular processes. Also, the role of water in the mass transport of ions through confined geometries was discussed. The interfacial friction between planar and nanoscopic layers lubricated by water was shown to significantly affect ion correlations and electroosmotic flow. Additionally, the confinement-induced structure of water was proven to influence processes like membrane desalination, osmotic power harvesting, and the deformation of elastic sheets under stress. We have extensively discussed, leading to a general improvement of our fundamental understanding: T1: Transport of charged species in bulk and confinement, T2: Charged active particles near charged surfaces, T3: Collective behavior of charged species in external electric fields, T4: The role of charges in nanopore DNA/RNA sensing, T5: Membrane deformation, permeation, budding, and encapsulation.

Concerning the first two subtopics T1 and T2, the discussions included the examination of the electric field generated at a water–air interface, where simulations were performed on 352 water molecules, over a time span of 200 psec (by Roland Netz). These effects were proven to play a role in the theoretical microscopic investigations of the electrophoretic mobility of charged colloids at high concentrations and electric conductivity (by H. Orland). Also discussed was the swelling/deswelling behavior of microgels, governed by electrostatics and elasticity, as well as the resulting structural and dynamics (by G. Naegele and A. Denton). Electrostatic interactions and charge regulation were highlighted as key factors in these processes, as well as in the behavior of inhomogeneously charged spherical particles (by A. Bozic). The discussion also extended to more complex macromolecular systems, such as ring polymers, which exhibit a distinct phase behavior and glass formation compared to linear polymers (by C. Likos).

Subtopic T3 overlapped considerably with T1 and T2 but focused more on the effects induced by external fields, such as the transport of charged particles in nonpolar media and the reversible formation of micelles under an electric field, and it was shown that micelles can exchange charges (by K. Neyts). The effects of external electric and magnetic fields, with varying frequencies, were further quantified using generalized Green–Kubo response functions in combination with simulations of electrolyte solutions (by B. Rotenberg). Additionally, quadrupolar nuclear magnetic resonance relaxation experiments were conducted to investigate the response of electrolytes in aqueous solutions to frequency-dependent fields (by I. Chubak). Frequency-dependent impedance measurements of nanocapacitors (by G. Pireddu) and electroacoustic spinning experiments (by T. Saghaei) were also presented.

As an introduction to subtopics T4 and T5, a theoretical analysis of the origin of attractive electrostatic interactions between like-charged surfaces with mobile charges was presented (by E. Trizac). Interactions between non-uniformly charged colloids were discussed (by M. Bier), and the dielectric polarizability of polyelectrolytes in electrolyte solutions was addressed (by F. Schmid). Subsequently, the role of charges in nanopore DNA/RNA sensing was presented, based on the MARTINI program (by G. Settanni). Also, the electro-permeability of hydroperoxide lipid membranes was discussed (by C. Marques) and results from an experimental investigation using angle-resolved XPS were presented, focusing on the microstructural order and anisotropic diffusive transport of ions in supported ionic liquid phases (by A.-S. Smith).

Furthermore, we also discussed the physics of active particles, including synthetic particles, bacteria, sperm, and others. An approach to describe the interactions between active Brownian particles was presented, drawing an analogy with electrostatic interactions between inactive charged colloids (by B. Liebchen). Additionally, the behavior of a single active Brownian particle embedded in a viscoelastic fluid, with small inertial forces, within a microfluidic channel was explored. A comprehensive analytical theory was presented to capture this complex behavior (by H. Stark)

## Geographical distribution of participants

The list below organizes the participants of the series of workshops according to their country of affiliation, with names followed by their institutional associations.

*Austria* Emanuela Bianchi (TU Wien), Christos Likos (University of Vienna), Gerhard Kahl (TU Wien)

*Belgium* Kristiaan Neyts (Ghent University), Filip Strubbe (Ghent University)

*France* Sam Hanot (Institut Laue-Langevin), Henri Orland (CEA Paris-Saclay), Benjamin Rotenberg (CNRS, University Pierre and Marie Curie, Sorbonne Université), Iurii Chubak (Sorbonne Université), Emmanuel TRIZAC (Université Paris-Sud), Carlos Marques (CNRS Strasbourg, Ecole Normale Supérieure de Lyon)

*Germany* Vania Calandrini (IAS-5/INM-9 Forschungszentrum Jülich), Paolo Carloni (IAS-5/INM-9 Forschungszentrum Jülich), Alexey Eremin (University of Magdeburg), Christoph Gögelein (LANXESS Deutschland GmbH), Nicolas Rivas (IEK-11 Forschungszentrum Jülich), Gunnar Schröder (ICS-6 Forschungszentrum Jülich), Jan-Philipp Machtens (ICS-4 Forschungszentrum Jülich), Jens Harting (HIERN, Forschungszentrum Jülich), Jerome Crassous (Lund University, RWTH-Aachen), Gerhard Naegele (IBI-4 Forschungszentrum Jülich), Roland Netz (Free University Berlin), Anselm Horn (Friedrich-Alexander-Universität Erlangen-Nürnberg), Frank Schreiber (University of Tübingen), Friederike Schmid (Johannes Gutenberg University Mainz), Holger Stark (TU Berlin), Maximilian Becker (Free University Berlin), Ana-Suncana Smith (Universität Erlangen), Benno Liebchen (Technische Universität Darmstadt), Jürgen Horbach (Heinrich Heine University Düsseldorf), Markus Bier (Max Planck Institute for Intelligent Systems), Claude Oelschlaeger (KIT), Giovanni Settanni (Univ. of Mainz, Ruhr Univ. Bochum), Kyongok Kang (IBI-4 Forschungszentrum Jülich), Jan K. G. Dhont (IBI-4 Forschungszentrum Jülich)

*Japan* Hitoshi Washizu (University of Hyogo), Akira Onuki (Kyoto University)

*South Korea* Zang-Hee Cho (Seoul National University), Do Hyun Kim (Korea Advanced Institute of Science and Technology)

*The Netherlands* Jens Harting (Eindhoven University), Nick Tasios (Utrecht University), Ben Werkhoven (University of Utrecht), René van Roij (Utrecht University)

*Slovenia* Anze Bozic (Josef Stefan Institute Ljubljana)

*Sweden* Jan Forsman (Lund University), Malin Zackrisson Oskolkova (Lund University)

*Switzerland* Peter Fischer (ETHZ Zurich)

*United Kingdom* Mira Zorkot (University of Oxford), Jure Dobnikar (University of Cambridge, Beijing University China)

*USA* Alan Denton (North Dakota State University), Daniel Hammer (University of Pennsylvania), Alpha Lee (Harvard University), Gerald S. Manning (Rutgers University), Orlin D. Velev (North Carolina State University)

## Tribute to the co-organizers: Gerhard Kahl and Jan Dhont

We would like to conclude our editorial and the series of CECAM Workshops by expressing our gratitude to Gerhard Kahl and Jan Dhont for their support, through a brief overview of their careers.

*Prof. Gerhard Kahl* was born in 1957 in Vienna, a city renowned for nurturing many brilliant minds that influenced modern culture. Inspired by this intellectual legacy, Gerhard Kahl decided to study physics, enrolling in 1975 at Technische Universität Wien, where he completed his doctoral studies in 1983 under the supervision of J. Hafner. Gerhard Kahl demonstrated academic excellence from a young age and maintained it throughout his studies, a distinction recognized by the prestigious “Promotio sub auspiciis Praesidentis rei publicae.” This honor, which includes a ring presented by the President of Austria, is a special form of doctoral graduation awarded to students who achieve the highest marks in every exam from primary school through to university. After completing his doctoral studies, Gerhard Kahl spent a brief period of research in Vienna supported by the Ludwig Wittgenstein Scholarship and then moved to Paris on an Erwin Schrödinger Fellowship for a postdoctoral position under the supervision of Jean-Pierre Hansen, a pillar in liquid state physics. However, Austria was not willing to let its brilliant mind slip away, and he returned to his home country, where he became assistant (1988), associate (1997) and university professor (2011) at the Institut für Theoretische Physik of the Technische Universität Wien. In 2007, shortly after the establishment of the CECAM node in Vienna (DaCAM), he became its director. Under his leadership, DaCAM has become a leading center for interdisciplinary research, specializing in computational methods in physics, chemistry, and materials science. Gerhard Kahl’s research covers a wide range of topics, including the self-assembly and phase behavior of complex colloidal systems (such as patchy and inverse patchy colloids, DNA-dendrimers, and microgels), as well as various computational methods; these include density functional theory and evolutionary algorithms for predicting phase behavior and self-assembly patterns in soft matter systems, particularly in confined environments and under external fields (such as shear, electric, and magnetic fields). His passion for research, which led him to publish cornerstone papers in various branches of soft matter, is matched only by his dedication to teaching: Gerhard inspired multiple generations of young minds, mentoring many talented students at the beginning of their scientific careers. His influence extended beyond academia: trained as a high-school teacher, he taught in high school both at the start of his scientific careers and even after his official retirement from the university, instilling a passion for physics in teenagers and inspiring bright minds to pursue science.

*Prof. Jan K. G. Dhont* completed his PhD thesis on multiple light scattering in 1985 at the van’t Hoff laboratory, University of Utrecht, The Netherlands under the supervision of Prof. A. Vrij. After a subsequent two-year Post Doc at the University of Konstanz in the group of Prof. R. Klein, he was appointed as an associated professor at the same van’t Hoff laboratory, in the group of Prof. H.N.W. Lekkerkerker in 1987. In 2000, Jan Dhont was appointed as a director at the Research Center of Juelich (FZJ), Germany, and in addition in 2001 as a full professor at the Heinrich-Heine-University in Duesseldorf, Germany. His scientific interests are mainly concerned with diffusion of several types of colloids in equilibrium, phase separation kinetics, as well as the response of colloidal suspensions to external fields, like shear flow, confinement, electric fields, and temperature gradients, both experimentally and theoretically. He wrote an introductory textbook in 1996 on some of these topics with the title “An Introduction to dynamics of Colloids,” published by Elsevier. Since 2000, together with colleagues from the FZJ (Prof. G. Gompper and Prof. D. Richter), he organized the annual Juelich Soft Matter Days (JSMD), a series of conferences dedicated to a multitude of aspects of soft matter systems in general. Some years later, Jan Dhont was involved in the organization of a larger, more internationally aimed conference, the International Soft Matter Conference (ISMC), which now takes place alternately in Europe, Asia, and the USA. This conference originated from an FP6 European network SoftComp (Soft Matter Composites) that was initiated by Prof. D. Richter. This network significantly gained momentum over the years, without funding from the EU. The EU infrastructure EUSMI originated from SoftComp as well, of which he was the coordinator, with Prof. P. Lang as the manager, who kept this project alive over the last few years as part of a larger EU project RIANA (Research Infrastructure Access in Nanoscience & Nanotechnology). Jan Dhont was appointed to the editorial board of the journal Soft Matter in 2016 for six years and received an honorary doctor from the University of Lund, Sweden, in 2021. Since his official retirement in October 2022, he is as before scientifically active in the FZJ. Jan Dhont inspired and guided many young scientist during their study and scientific career. In doing so, he is always emphasizing the great virtue of combining theory and experiments (of course in proportion, being either an experimentalist or a theoretician). A true understanding of physics includes most importantly a thorough intuitive explanation of complex phenomena.
